# CHEK2 mutations as markers for high risk of breast cancer

**DOI:** 10.1186/1897-4287-10-S3-A4

**Published:** 2012-04-20

**Authors:** Cezary Cybulski, Dominika Wokołorczyk, Anna Jakubowska, Tomasz Huzarski, Tomasz Byrski, Jacek Gronwald, Tadeusz Dębniak, Bohdan Górski, P Blecharz, S A Narod, Jan Lubiński

**Affiliations:** 1 Pomeranian Medical University, Clinic of Oncology, Szczecin, Poland; 2 Oncology Institute, Kraków, Poland; 3 Women’s College Research Institute, Toronto, Ontario, Canada

## 

Genetic testing for the two major breast cancer susceptibility genes, BRCA1 and BRCA2 is widely available in North America and Europe. A few other highly-penetrant breast cancer genes have been found, including p53, BRIP1 and PALB2, but families with mutations in these are exceedingly rare. Arguably, the most relevant of the post-BRCA genes, from a clinical point of view, is CHEK2, which was first linked to breast cancer susceptibility in 2002.

In Poland, there are four founder mutations of CHEK2. Three of these (IVS2+1G>A, del5395 and 1100delC) are protein-truncating mutations and one (I157T) is a missense variant. We estimated the lifetime risk of breast cancer for carriers of CHEK2 truncating mutations to be 20% for a woman with no affected relative, 28% for a woman with one second-degree-relative affected, 34% for a woman with one first-degree relative affected, and 44% for a woman with both first- and one-second degree relative affected in the Polish population. In addition we estimated that the lifetime risk for breast cancer for women who carried two different CHEK2 mutations (a truncating mutation and the missense mutation) to be 42%.

Our results confirm that CHEK2 mutation screening detects a clinically meaningful risk of breast cancer, and women with a truncating mutation in CHEK2 and a positive family history of breast cancer, and women who carry two different CHEK2 mutations (the missense mutation I157T and a truncating mutation) face a lifetime risk of breast cancer above 25% and are candidates for MRI screening and for tamoxifen chemoprevention.

